# The effects of SGLT2 inhibitors on metabolic phenotype and FGF-21 expression from the adipose tissue and the liver are less pronounced in *ob*/*ob* mice

**DOI:** 10.1186/s12902-025-01879-3

**Published:** 2025-03-10

**Authors:** Angelo Di Vincenzo, Marnie Granzotto, Marika Crescenzi, Paola Fioretto, Roberto Vettor, Marco Rossato

**Affiliations:** 1 Department of Medicine, University of Padua, Padova, Italy; 2 Unit of Internal Medicine 3, University Hospital of Padova, Padova, Italy; 3 IRCCS Humanitas Research Hospital, Rozzano, Milan, Italy

**Keywords:** Genetic obesity, Adipose tissue, Liver, SGLT2 inhibitor, FGF-21

## Abstract

**Background:**

the metabolic effects of sodium-glucose cotransporter-2 inhibitors (SGLT2i), such as lipolysis and ectopic fat reduction, seem related to the synthesis of fibroblast growth factor-21 (FGF-21), and FGF-21 analogs are now under investigation for the treatment of obesity complications such as metabolic dysfunction-associated steatotic liver disease. However, FGF-21 levels are paradoxically higher in obesity, indicating a hormone-resistant state that may hinder the benefits of SGLT2i.

**Methods:**

To define if a different energy status influences the response to SGLT2i, we evaluated the effects of dapagliflozin administration on nine-week-old C57BL/6J wild-type and B6.V-LEP *ob*/*ob* mice as a model of genetic obesity. Blood glucose, body weight and food intake were evaluated, and the FGF-21 expression was determined in subcutaneous adipose tissue (SAT), visceral adipose tissue (VAT), and brown adipose tissue (BAT). In the liver, FGF-21 gene expression, protein concentration and triglyceride content were evaluated.

**Results:**

glucose plasma levels and body weight were higher in *ob*/*ob* than in lean mice. After four weeks of treatment, dapagliflozin reduced blood glucose levels and body weight in both animal models, but weight loss was more significant in lean mice. The baseline expression of FGF-21 was higher in both SAT, VAT and the liver of *ob*/*ob* mice, whereas it was almost undetectable in BAT in both animal groups. After the treatment period, dapagliflozin was shown to increase FGF-21 expression in VAT only in lean animals, while the expression was unaffected in *ob*/*ob* mice. Similar effects were observed in the liver analyses, along with no variation in triglyceride content.

**Conclusions:**

SGLT2i administration results in less pronounced metabolic effects in *ob*/*ob* mice than in lean mice. This data suggests a less sensitive response in obesity, probably due to a chronic stimulation leading to abnormalities of the SGLT2i-FGF-21 axis which should be considered in managing patients affected by genetic obesity.

## Background

Since their introduction, sodium-glucose cotransporter-2 inhibitors (SGLT2i) have displayed benefits that go beyond their anti-hyperglycemic action. These benefits have been confirmed by several large clinical trials encouraging their use in treating conditions other than type 2 diabetes mellitus (T2DM) such as heart failure and chronic kidney disease [1, 2], and now SGLT2i seem also promising in slowing the progression of metabolic dysfunction-associated steatotic liver disease (MASLD) [3]. However, the underlying mechanism for their protective effects cannot be attributed solely to the increased urinary glucose excretion. The evidence that SGLT2i treatment induces some degree of body weight loss with a reduction of ectopic fat deposition led to the hypothesis of a possible modulation of energy homeostasis [4], with SGLT2i inducing various biochemical alterations activating nutrient-deprivation pathways in target organs [5], or promoting the activation of non-shivering thermogenesis Of note, it remains to be established if these modifications are displayed only in conditions of energy excess or are maintained even in the absence of energy surplus and large excess of adiposity.

Accumulating evidence proposes a role for endogenous molecules in the modulation of the glycemic-independent actions of SGLT2i and among them, the fibroblast growth factor 21 (FGF-21) seems to have a prominent role. FGF-21 is a protein with paracrine and endocrine properties [6], whose biological activity lies in the interaction with cell surface FGF-related receptors (FGF-Rs) and the adaptor molecule *β-*klotho [7]. FGF-21 presents a multifaceted metabolic profile as it is involved in adipose tissue lipolysis [8, 9], and hepatic lipid content reduction after SGLT2i treatment [10], but surprisingly patients with obesity and T2DM show higher circulating levels of FGF-21 than expected [11, 12], thus questioning the real contribution of FGF-21 to the overall SGLT2i benefit.

To investigate whether different energy statuses affect the response to SGLT2i, we investigated the metabolic effects of dapagliflozin in lean and a genetic model of obesity represented by *ob*/*ob* leptin-deficient mice. We measured changes in body weight, food intake, and liver triglyceride content along with the expression of FGF-21 to determine the impact of the treatment.

## Methods

### Animals and experimental procedures

#### Ethics statement

The animal care procedures followed institutional guidelines in compliance with national and international laws and policies (European Economic Community Council Directive 86/609, OJ L 358, 1 Dec.2012, 1987; NIH Guide for the Care and Use of Laboratory Animals, NIH Publication no. 85 − 23, 1985). The study design was approved by the Ethics Committee of the University of Padova for the use of animals (CEASA protocol number 58/2018-PR, approved by the Ethics Committee for animal studies (OPBA) of the University of Padova). The study was finally approved by the Italian Ministry of Health (protocol n. 58/2018-PR). We complied with ARRIVE (Animal Research: Reporting of In Vivo Experiments) guidelines for all the experiments.

#### Murine models and experimental procedures

**Fig. 1:**
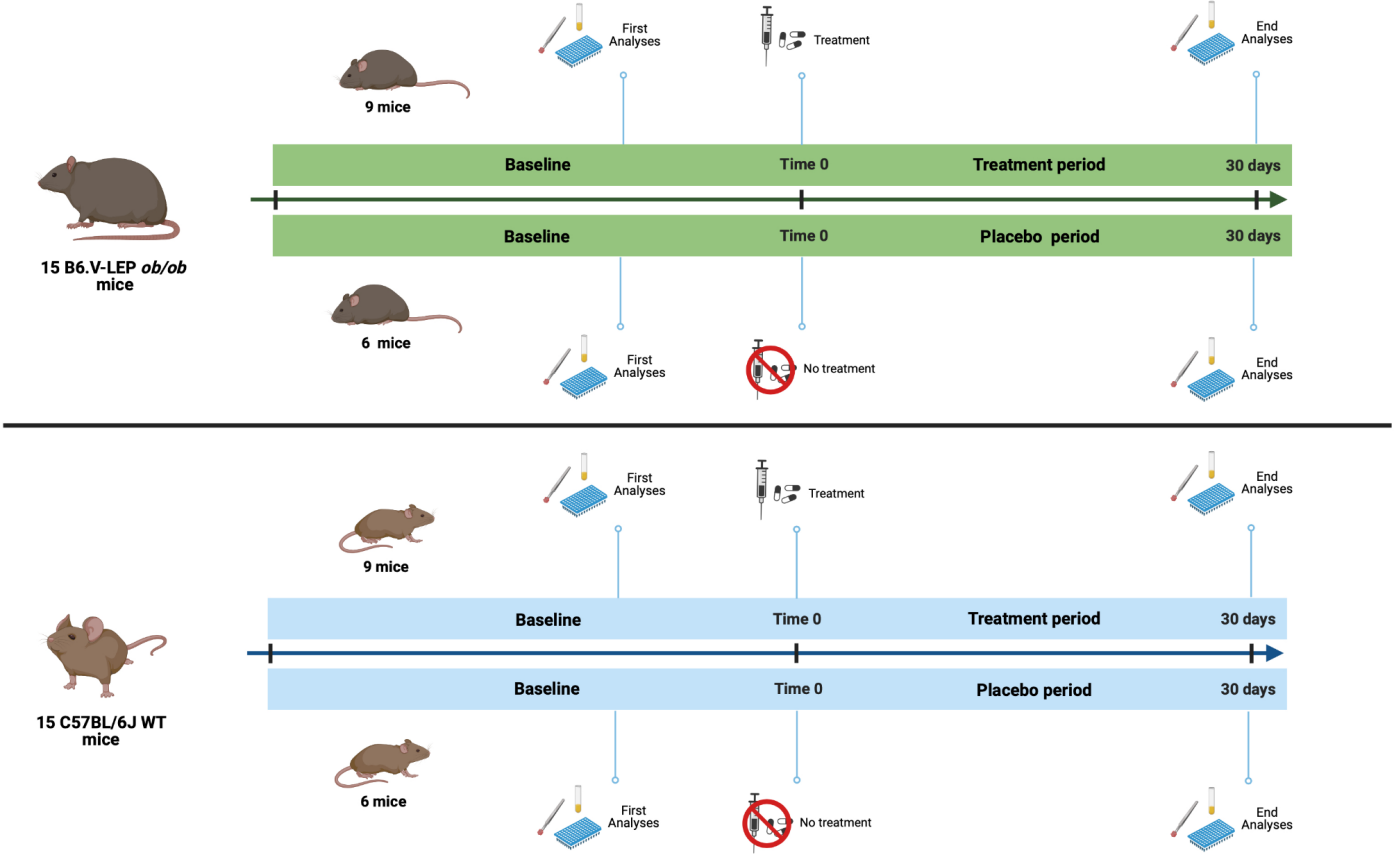
Graphical representation of the study design

We performed these experiments in the context of a more extensive research project with different aims published elsewhere [13]. A graphic representation of the study design is reported in Fig. 1. We used nine-week-old (acquired 6-week-old mice from the vendor, then acclimated for 20 additional days) C57BL/6J wild-type (WT) mice and B6.V-LEP *ob*/*ob* mice obtained from Charles River Laboratories International Inc., Wilmington, Massachusetts, USA. The mice were housed in a pathogen-free and temperature-controlled environment, with a 12-hour light/dark cycle, and were given access to a chow diet and water *ad libitum*. According to our study design, we obtained 15 WT and 15 *ob*/*ob* mice: for each type group, 6 animals were managed only with a chow diet (soaked treat with lactose) serving as control, while 9 mice in addition to the chow diet underwent treatment based on the administration of dapagliflozin 0.15 mg/Kg/day (according to the producer the concentration more similar to clinical use) via treats soaked for 4 weeks. Baseline and end-of-study measurements were recorded for body weight and blood glucose. The determination of glycemia was performed by means of a glucometer on whole blood after caudal vein puncture under fasting conditions. Food intake and food efficiency (defined as the ratio between weight gain and food intake) were evaluated after 15 days and at the end of the treatment. At the end of the treatment and after performing the experiments, the animals were sacrificed with an intraperitoneal overdose of Zoletil (tiletamine/zolazepam 200 mg/kg) and then euthanized through cervical dislocation.

### RNA extraction and real-time PCR

Experiments were conducted according to our standardized method. Total RNA was extracted from the liver, subcutaneous, visceral, and brown adipose tissue, using the RNeasy Tissue mini kit (QIAGEN, Milan, Italy). RNA content was quantified using a DS-11 spectrophotometer (DeNovix Inc.3411 Silverside Rd. Wilmington DE USA). RNA samples (2 µg) were treated with DNase and Removal Reagents (Thermo Fisher, Waltham, MA, USA) and reverse-transcribed for 1 h at 37 °C with 150-ng random primers, 0.5-mM dNTPs, 20 units of RNAsin Ribonuclease Inhibitor and 200 units of M-MLV RT (Promega, Madison, WI, USA). The expression of mouse Fibroblast Growth Factor-21 (FGF21) FGF-21 receptors (FGFRs R1, R2, R4) and co-receptor *β*-klotho, fibrosis-related markers (TGF-β, α-SMA, PDGF-β, TNF-α) and energy expenditure marker UCP1, PGC1-α and CIDEA were assessed by Real-time PCR with SYBR™ Select Master Mix (Thermo Fisher). The primers used for real-time PCR quantification are reported in Table 1. The fluorescence change was measured at every cycle and a threshold cycle above the background was calculated for each reaction. Each reaction was carried out in duplicate. For the determination of the relative concentration, gene expression was normalized to β_2_-microglobulin calculated by the change-in-cycling-threshold method as 2^−ΔΔC(t)^. A melt curve analysis was conducted after each reaction to confirm the amplification of a single product.

**Table 1:** Primers used in real-time PCR quantification

Gene name		Sequence	Amplicon bp
*Fgf21*	F	CCGCAGTCCAGAAAGTCTCC	144
	R	CTGCAGGCCTCAGGATCAAA	
*Fgf_receptor1*	F	AAGGACAAACCCAACCGTGT	148
	R	ACGCTCCCAGAAGGTTGATG	
*Fgf_receptor2*	F	TTGCCGAATGAAGACCACGA	100
	R	AACTGTTACCTGTCTCCGCA	
*Fgf_receptor4*	F	CTTTGGGCAAGTGGTTCGTG	108
	R	TCCTTGTCGGAGGCATTGTC	
*Klb*	F	CACAACCTGATCAAGGCACATT	100
	R	AATGGGACCCCAAGGTGATG	
*Cidea*	F	TCTTGGAAAGGGACAGAAATGGA	142
	R	TTGACATTGAGACAGCCGAGGA	
*Pgc1a*	F	CCACTACAGACACCGCACACACC	145
	R	TTCATCCCTCTTGAGCCTTTCG	
*Ucp1*	F	CCTGCGGGCATTCAGAGGCAA	161
	R	TGCCCAATGAACACTGCCACA	
*TNFa*	F	CTTCTGTCTACTGAACTTCGGG	108
	R	CAGGCTTGTCACTCGAATTTTG	
*α_SMA*	F	AGCCATCTTTCATTGGGATGGAG	128
	R	CATGGTGGTACCCCCTGACA	
*PDGFβ*	F	CCGGTCCAGGTGAGAAAGAT	184
	R	GAATGGTCACCCGAGCTTGA	
*TGF β*	F	GCCCGAAGCGGACTACTATG	139
	R	CACTGCTTCCCGAATGTCTG	

### FGF-21 protein determination

The tissues were minced and then covered with a cold lysis buffer containing 50-mM HEPES, pH 7.5, 150-mM NaCl, 10% glycerol, 5-mM Triton-X-100 and protease/phosphatase inhibitor cocktails (Calbiochem, Darmstadt, Germany and Sigma-Aldrich, St. Louis, MO, USA), and homogenized with Tissue Lyser II (Qiagen) for 3 min, then extracts were centrifuged and protein concentration was determined in the supernatants by the colorimetric Bradford method. A Sandwich High Sensitivity FGF-21 ELISA kit (EK 5641, Sabbiotech) was utilized to detect mouse FGF-21 with a 96-well strip plate.

### Hepatic triglycerides measurement

To determine the concentration of triglycerides in the liver, the colorimetric assay Triglyceride Quantification Kit (ab65366, Abcam) was used. In brief, 100 mg of liver tissue was homogenized in a Tissue Lyser II (Qiagen) with 1 mL of 5% NP-40/ddH2O solution. The homogenate was then heated slowly to 80–100 °C and kept in a water bath for 5 min to solubilize all triglycerides. The enzyme mix was used to convert triglycerides to free fatty acids (FFA) and glycerol. Glycerol was then oxidized to generate a product that reacted with a probe to create color (570 nm).

### Staining

The frozen tissues were cryostat-sectioned and then stained with Oil-red-O (ORO; Sigma cat. No. #O-0625). To prepare the solution, 30 mL of ORO stock were mixed with 20 mL of distilled water, and the mixture was filtered. The air-dried frozen sections were fixed in neutral buffered formalin for 10 min, washed in 60% isopropanol, and stained with the working ORO solution for 15 min. Next, the sections were dipped in 60% isopropanol, followed by distilled water, and counterstained with Mayer’s hematoxylin for 3 min. The sections were then incubated in tap water for 10 min and mounted with aqueous mounting gel.

### Statistical analysis

Statistical analysis was performed with GraphPad Prism version 6 for Windows (GraphPad Software, CA). Results are reported as means + SEM. Two-sided t-tests or Mann-Whitney were used to test the statistical significance of mean differences between two groups, while for multiple comparisons, ANOVA was performed, while the Kruskal-Wallis test was used when data did not show normal distribution. *p* values < 0.05 were considered statistically significant.

## Results

### Dapagliflozin administration improves metabolic phenotype in lean more than in *ob*/*ob* mice

**Fig. 2:**
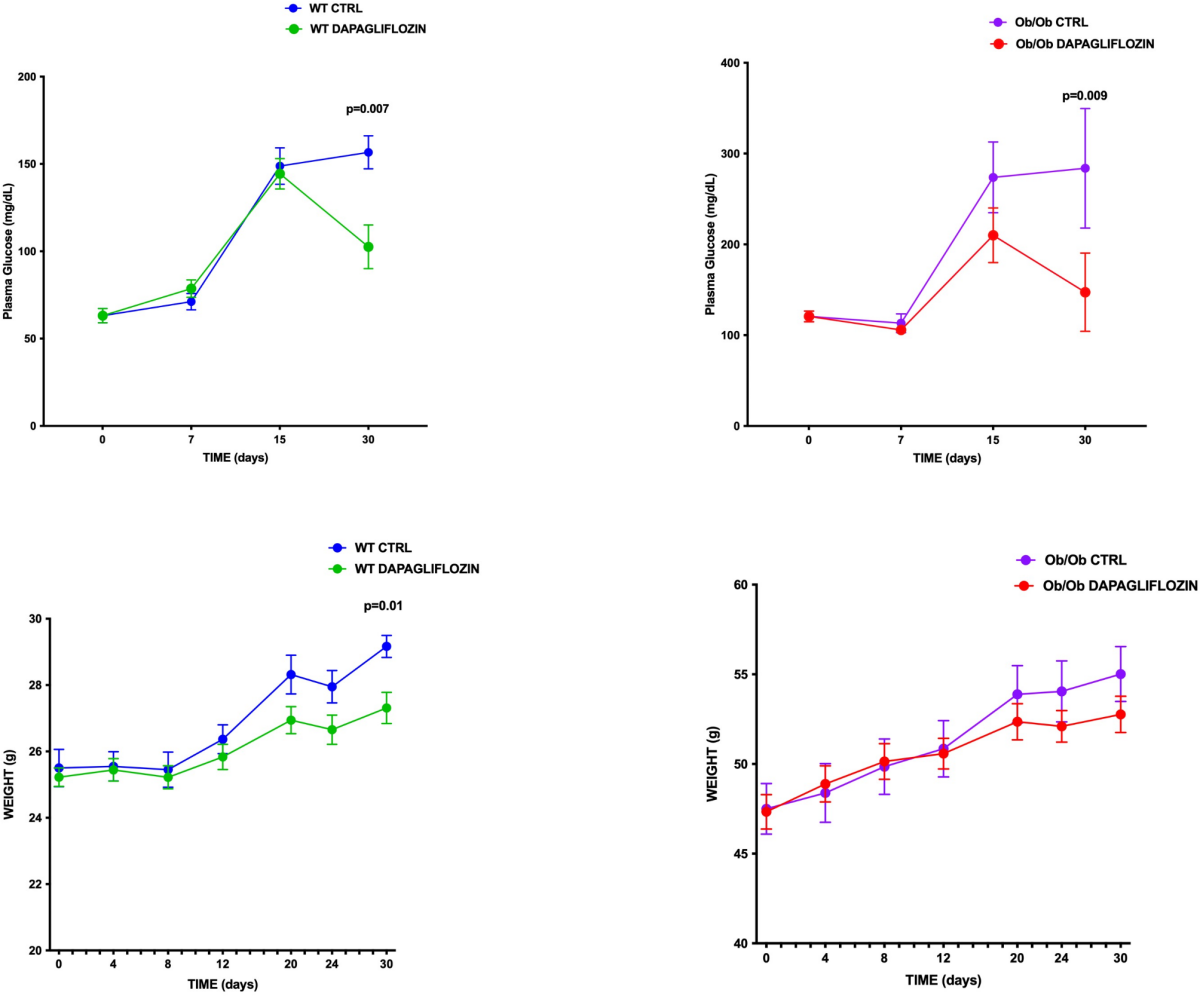
Effects of dapagliflozin administration on metabolic parameters in lean and *ob*/*ob* mice. Effects of dapagliflozin on blood glucose levels and weight trend analysis after treatment. After 30 days of treatment, a significant reduction of blood glucose plasma levels was observed in both lean and *ob*/*ob* animals treated with dapagliflozin. Body weight reduction was reported in both lean and *ob*/*ob* mice; however, the statistical significance was observed only in the treated lean group

**Fig. 3:**
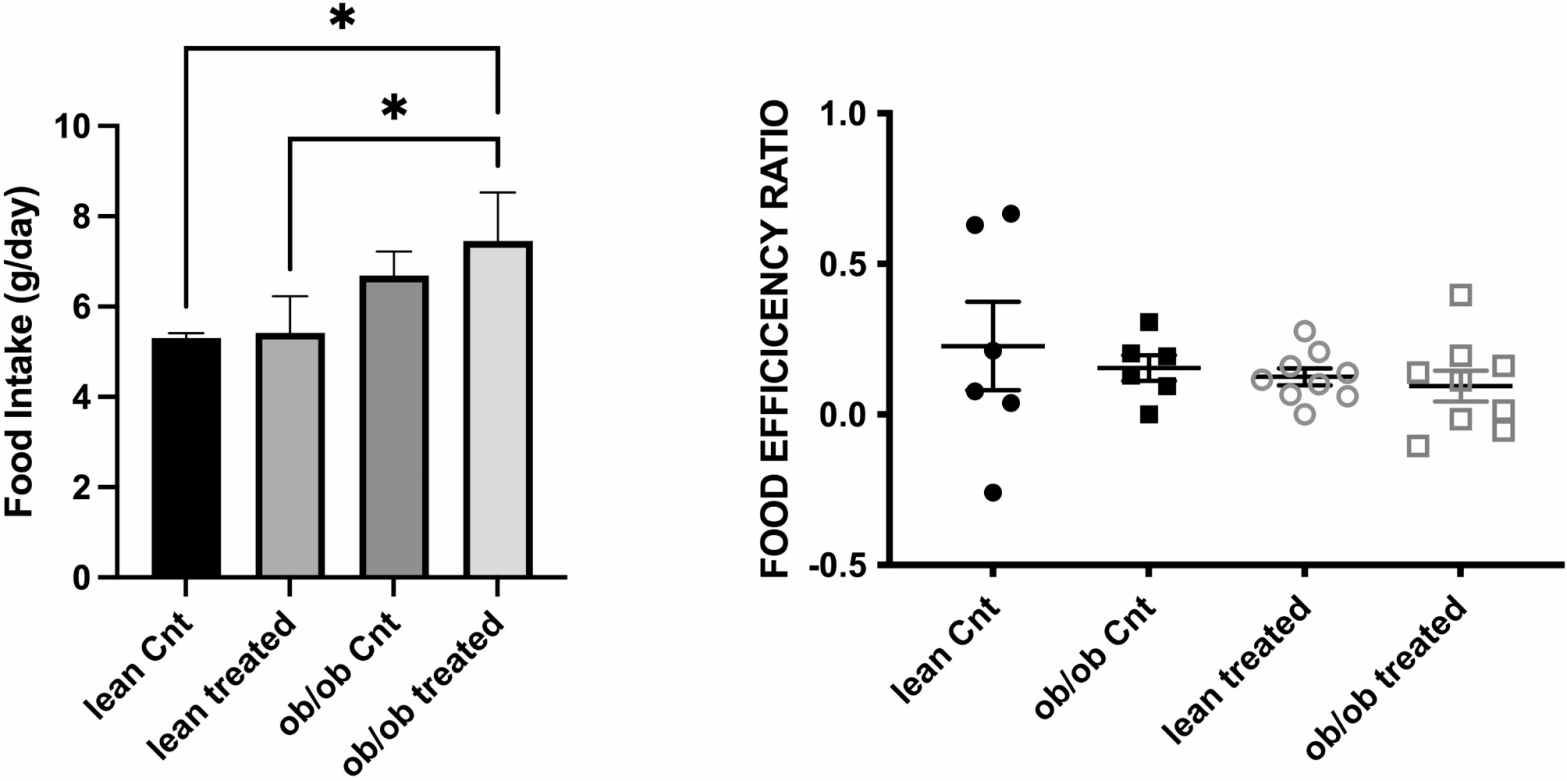
Evaluation of food intake and food efficiency in lean and *ob*/*ob* mice before and after dapagliflozin administration. At baseline, food intake was slightly higher in *ob*/*ob* with respect to wild-type animals. After treatment, a significant increase in food intake was observed in *ob*/*ob* mice with respect to lean mice, without a significant modulation of food efficiency. Cnt: no treatment group *p*0.01)

**Fig. 4:**
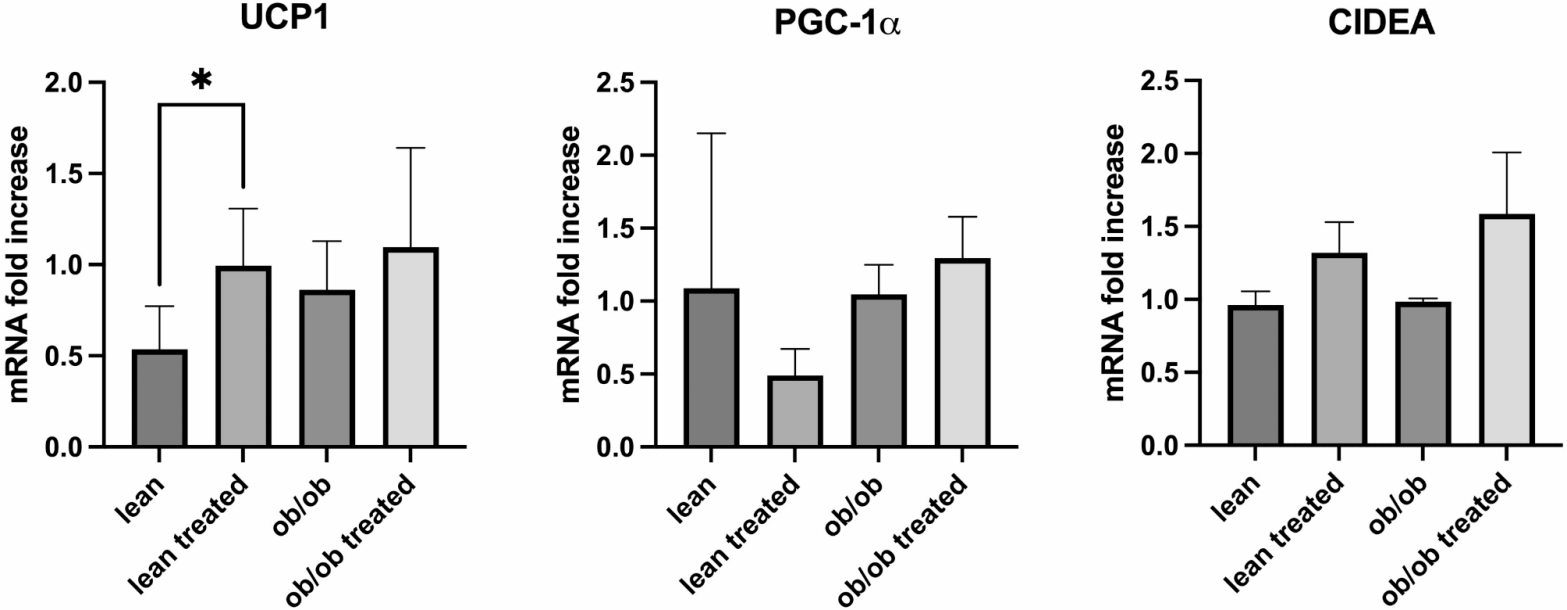
Effects of dapagliflozin administration on markers of BAT activation. Considering the potential modulation of FGF-21 on thermogenesis markers of BAT activation (UCP-1, PGC-1α, CIDEA) were evaluated. Of note, dapagliflozin treatment significantly increased mRNA expression of UCP-1 in lean animals but not in *ob/ob* mice (^*^p < 0.05)

As expected, blood glucose levels significantly differed at baseline between lean and *ob*/*ob* mice (63 vs. 125 mg/dL, *p* < 0.05); while a significant reduction of glycemia was observed in both lean and *ob*/*ob* animals after treatment with dapagliflozin (100 vs. 155 mg/dL, *p* = 0.007, and 156 vs. 280 mg/dL, *p* = 0.009 respectively, Fig. 2A). Obviously, from time zero till the end of the study, the animals fed *ad libitum* increased their body weight compared to the baseline; however, we also observed that the administration of dapagliflozin led to an overall reduction of body weight that reached statistical significance only in lean animals (55 g *ob*/*ob* control vs. 52 g *ob*/*ob* treated, *p* = 0.9, lean control 29 g vs. 26.8 g lean treated, *p* = 0.01, Fig. 2). We also evaluated the food intake trend during the study. We observed at baseline only a small not statistically significant higher food intake in the hyperphagic *ob/ob* mice. In contrast, after dapagliflozin treatment, a significant increase in food intake was observed in *ob*/*ob* animals but not in treated lean mice, and without evident modification of efficiency ratio (Fig. 3). To determine whether SGLT2i treatment affected BAT activation, justifying these results, we evaluated markers of thermogenesis in BAT. Notably, dapagliflozin treatment significantly increased UCP1 expression in lean animals compared to *ob/ob* mice, potentially explaining the difference in body weight reduction observed between the two models (Fig. 4).

### Dapagliflozin administration enhances FGF-21 gene expression in lean but not *ob*/*ob* mice

**Fig. 5:**
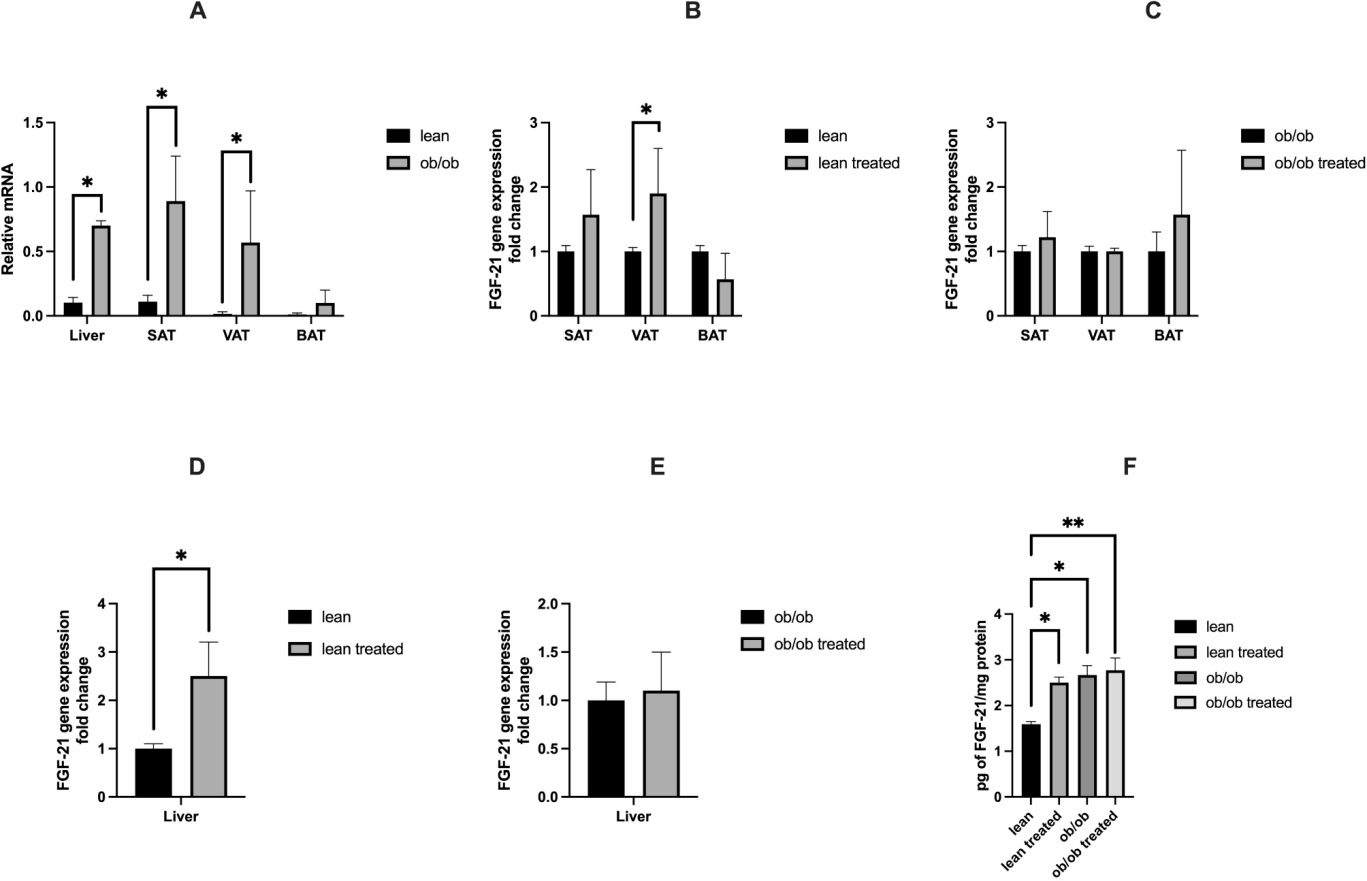
Baseline expression of FGF-21 and the effects of dapagliflozin administration in lean and *ob*/*ob* mice. **A**. At baseline, higher expression of FGF-21 was found in *ob*/*ob* compared to lean mice in all considered tissues except BAT. **B** and **C**. Effects of dapagliflozin treatment on FGF-21 expression in SAT, VAT and BAT in *ob*/*ob* and lean mice. Analysis of FGF-21 mRNA expression in lean and *ob*/*ob* mice following administration of dapagliflozin showed that the SGLT2i administration was associated with a significant increase in gene expression (expressed as fold change) in VAT of lean mice (**B**), whereas gene expression was unaffected by the treatment in *ob*/*ob* mice (**C**). **D**, **E** and **F**. Dapagliflozin affects hepatic FGF-21 only in lean mice. The SGLT2i administration promoted a significant increase in liver FGF-21 gene expression (**D** and **E**) and protein concentration (**F**) in lean but no *ob*/*ob* mice. BAT: brown adipose tissue; SAT: subcutaneous adipose tissue; VAT: visceral adipose tissue. ^*^p < 0.05; ^**^p < 0.01*

Since FGF-21 is considered the main mediator of pleiotropic effects of SGLT2i, we sought to test if dapagliflozin treatment affects the expression of FGF-21 in metabolically active tissues such as the liver, SAT, VAT, and BAT of both lean and *ob*/*ob* mice. At basal conditions, significantly higher expression of FGF-21 was observed in *ob*/*ob* mice with respect to lean animals in all the considered tissues except for BAT (Fig. 5A). Interestingly, treatment with dapagliflozin significantly affected the expression of FGF-21 in lean mice. In particular, we observed an increase in hormone gene expression in the VAT and the liver of lean mice (5B and 5D) without a further increase in *ob*/*ob* mice (5 C and 5E). A similar trend was observed in liver protein expression (5 F).

### Dapagliflozin treatment does not affect FGF21-related receptors expression

**Fig. 6:**
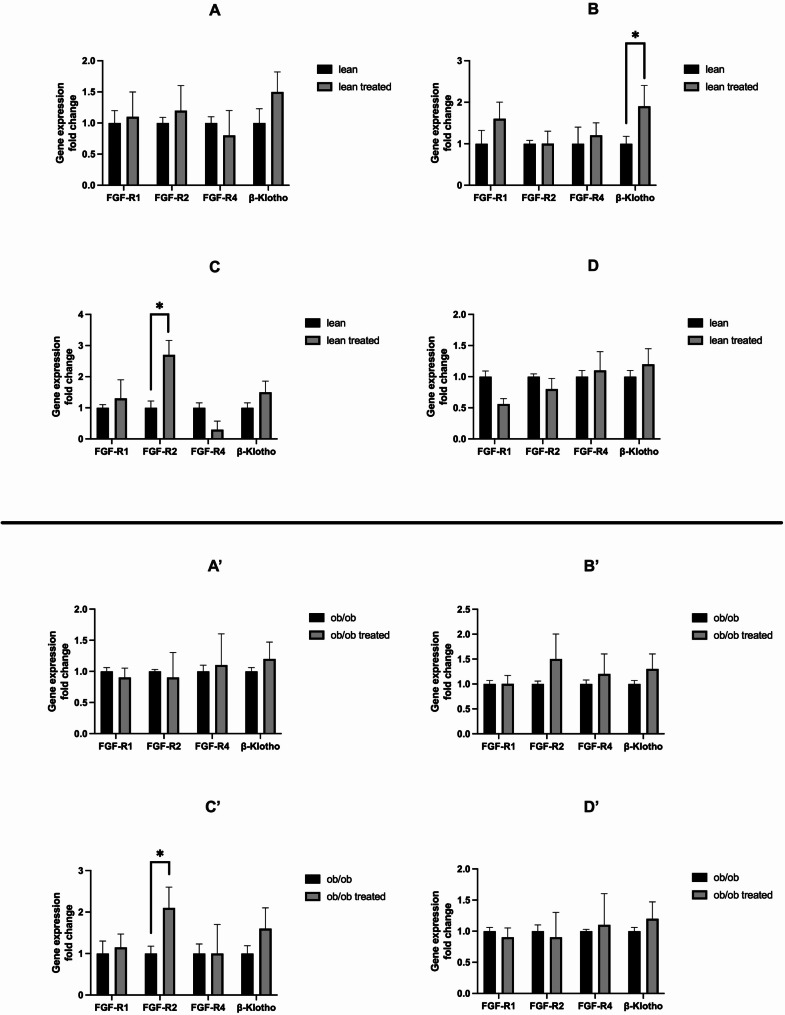
FGF-21 receptors expression analysis following dapagliflozin administration in lean and *ob*/*ob* mice. FGF-R1, FGF-R2, FGF-R4 and *β*-klotho gene expression was evaluated in the subcutaneous adipose tissue (**A** and **A**’), visceral adipose tissue (**B** and **B**’), brown adipose tissue (**C** and **C**’), and the liver (**D** and **D**’) in both lean and *ob*/*ob*. Data were expressed as fold change. ^*^p > 0.05

To test if dapagliflozin could affect FGF-21 receptors expression, we evaluated the expression of the FGF-R1, FGF-R2, FGF-R4, and *β*-klotho at baseline and after treatment. However, our findings suggest that dapagliflozin does not modulate the expression of FGF-Rs of the examined tissues in both lean or *ob*/*ob* mice (Fig. 6).

### Dapagliflozin treatment does not modify the liver triglyceride content in lean and *ob*/*ob* mice nor the expression of fibrosis markers

**Fig. 7:**
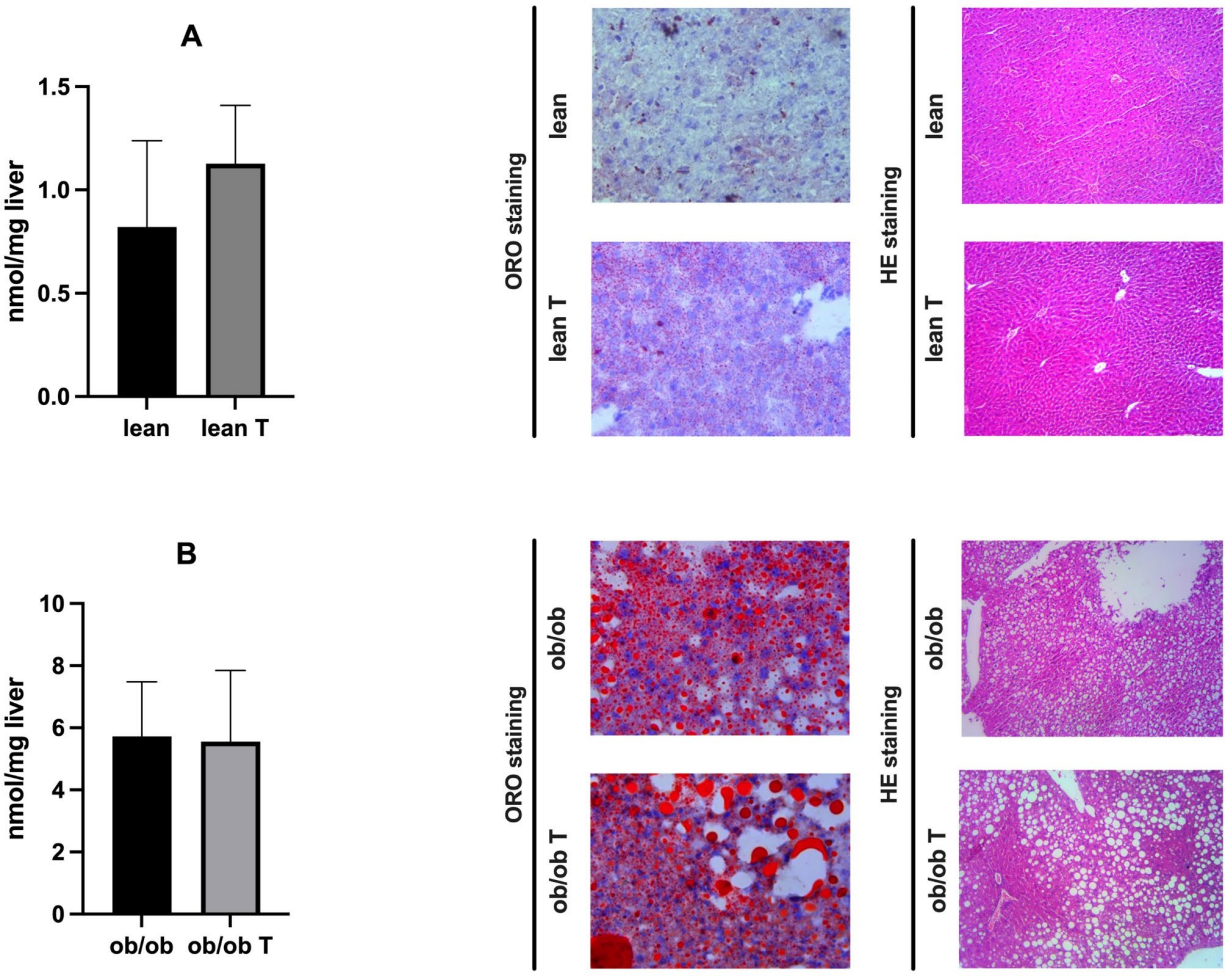
Dapagliflozin administration does not affect triglyceride content in both animal models. Triglycerides were measured in the liver of both lean (**A**) and *ob*/*ob* mice (**B**), and expressed as nmol/mg of tissue, before and after dapagliflozin administration. No significant differences were observed in lipid content after SGLT2i treatment. Representative ORO and HE staining images are reported next to the corresponding histogram (magnification x400)

**Fig. 8:**
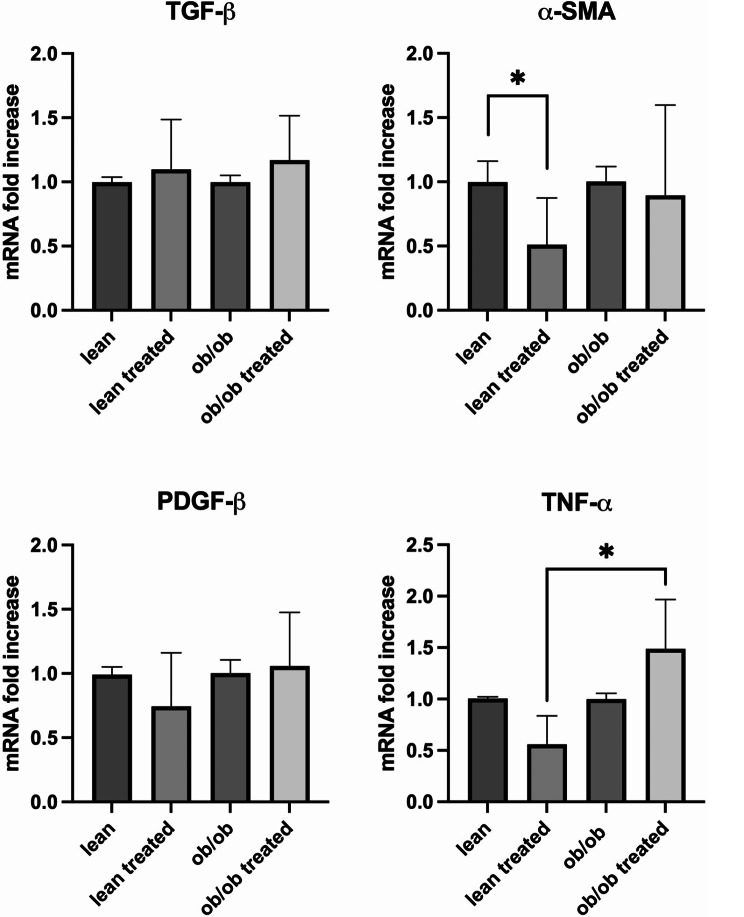
Dapagliflozin administration does not reduce the expression of liver fibrosis markers in *ob/ob* animals. TGF-β, α-SMA, PDGF-β, and TNF-α expression was evaluated in the liver of both lean and *ob/ob* mice. After treatment, a reduction of α-SMA was reported only in lean mice, while TNF-α even increased in *ob/ob* mice. Data were expressed as fold change. ^*^p > 0.05

Triglyceride content, expressed as nmol per milligram of liver tissue, was measured in lean and *ob*/*ob* mice before and after treatment with dapagliflozin. Macroscopic evaluation was performed using ORO and hematoxylin and eosin staining (HE). As expected, *ob*/*ob* mice had higher liver triglyceride levels than lean mice at baseline. Treatment with dapagliflozin did not affect triglyceride content in both lean and *ob*/*ob* mice. (Fig. 7). Additionally, we tested the effects of dapagliflozin on markers of hepatic fibrosis. Still, we did not report any coherent significant modifications, except for the TNF-α, whose expression even increased after treatment in ob/ob mice. In contrast, only α-SMA expression was reduced in lean mice (Fig. 8).

## Discussion

Despite the extensive use of SGLT2i in clinical practice, the mechanisms that underlie their benefits remain unclear. It is plausible that the promotion of lipolysis, the activation of fasting-mimicking cellular pathways, and the systemic release of FGF-21 contribute to their pleiotropic profile. However, which type of patients may experience greater efficacy, and if the whole activity of SGLTi is maintained in obesity, remains to be established. While studies have shown that the efficacy of SGLT2i remains consistent in patients with heart failure irrespective of obesity severity [14], other data reported that in hypertensive patients SGLT2i are less effective for BMI higher than 30 kg/m^2^ [15].

Herein, we confirmed the anti-hyperglycemic effect of SGLT2i in a genetic model of obesity represented by the *ob*/*ob* leptin-deficient mice, but we reported a reduction of blood glucose levels after treatment also in wild-type lean animals. Interestingly, we also reported a more significant body weight reduction after treatment in C57BL/6J mice with respect to B6.V-LEP *ob*/*ob* mice that experienced a higher increase in food intake after SGLT2i treatment. These results may suggest that some metabolic effects of SGLT2i may be maintained irrespective of baseline body energy status, or even be less effective in obesity.

SGLT2i administration is known to be associated with an increase in energy expenditure and a consequent compensatory increase in food intake, both modulated by variations in FGF-21 levels [16]. The release of FGF-21 is affected by a range of factors including physical activity, circadian rhythm, and dietary compounds [17], but also, as shown recently, SGLT2i. In addition, the relationship between SGLT2i and FGF-21 seems bidirectional, with FGF-21 indispensable for some SGLT2i-mediated effects such as adipose tissue lipolysis [10]. FGF-21 is produced by metabolic active tissues such as the liver but also the adipose tissue [18, 19], somehow explaining the supraphysiological hormone concentration and the impaired hormonal response in obesity defined as FGF-21 resistance state [20, 21]. With this in mind, we aimed to ascertain possible different effects of SGLT2i on the FGF-21 pathway in different energy statuses.

Firstly, we confirmed the obesity-associated up-regulation of FGF-21 expression in SAT, VAT, and the liver at baseline, but we also showed that SGLT2i treatment preferentially affects the expression of FGF-21 in lean more than in *ob*/*ob* mice. As mentioned before, FGF-21 expression is modulated by a variety of nutritional stimulants and inflammatory stressors to counteract the detrimental metabolic of energy excess. In this sense, adipocyte-derived FGF-21 acting in a paracrine manner may promote protective effects such as lipolysis and browning of white adipose tissue [22], leading to the positive effects of SGLT2i treatment. However, the elevation of FGF-21 plasma levels, starting as a protective mechanism, may promote a lower sensitivity to this hormone over time [23–25], and, in agreement with our results, even a reduced response to dapagliflozin treatment. We also took into account a possible downregulation of related FGF-Rs and *β*-klotho, whose reduction was already reported in both humans and *ob*/*ob* mice [26–28], but we did not observe a specific trend in FGF-Rs expression after SGLT2i treatment. In this context, it is important to note that the overexpression of β-klotho in adipose tissue does not appear to improve metabolic parameters or increase FGF-21 sensitivity in obese mice [29]. Due to the increased circulating levels of FGF-21, a higher concentration threshold of the hormone may be required in obesity for the activation of the related pathways, and this may explain the potential role of exogenous supplementation of the hormone as treatment for metabolic disease, as demonstrated by the results of phase II clinical studies showing benefits in ameliorating MASLD and adiposity [30]. We suppose that treatment with SGLT2i activates starvation-mimicking signalling leading to energy dissipation, including the release of FGF-21, but, in obesity, the extent of this response is limited and not sufficient to counteract the compensatory increase of food intake, thus limiting the drug potential on weight loss. Consistently, this may explain the increased UCP-1 expression reported in WT but not in *ob/ob* mice after treatment, with the activation of thermogenesis driving the more pronounced metabolic response to SGLT2i in lean animals. Certainly, at this point, our study lacks an instrumental evaluation for determining energy expenditure; additionally, to better understand the true nature of the FGF-21 resistance state, further mechanistic insights should be provided, particularly regarding modifications in the downstream signalling of the FGF-21 pathway in obesity, but in our opinion this intriguing hypothesis still merit to be further tested in the future.

Finally, we did not observe a significant reduction of liver triglyceride content after dapagliflozin treatment in both models. This is not so unexpected in lean animals, considering the small amount of lipids in their liver; on the contrary, in the *ob*/*ob*-treated mice, this result is not in line with what is reported in other basic and clinical studies, for which a potential for SGLT2i on MASLD has been reported [3]. It could be justified by the short duration of the therapy in our study, limited to four weeks, masking the potential benefits of the drug taking more time to develop its effect in reducing fatty liver content and mitigating insulin resistance, which is usually observed after longer treatment [31, 32]. However, other basic studies with a similar design showed hepatic steatosis improvement in mice with diet-induced obesity with the same treatment duration [33]. So, other conditions may account for the heterogeneity of the results among the different studies, such as the various animal models, the different severity of obesity, and concurrent metabolic abnormalities, as a standard model for MASLD is yet to be identified [34].

Furthermore, SGLT2i treatment did not significantly affect the expression of liver fibrosis markers, limiting their potential in liver fibrogenesis. However, we also should point out that studies evaluating the role of SGLT-2i in the treatment of MASLD often show conflicting results, in particular concerning histological outcomes, and current hepatological guidelines do not recommend SGLT2i administration outside usual indications [35].

Our research has some limitations. First of all, we acknowledge that our study is mainly descriptive, lacking mechanistic insights, and that we focused particularly on FGF-21 expression. In addition, somebody could argue about the use of a genetic model of obesity over a common diet-induced obesity model. *Ob/ob* mice are typically regarded as a reliable model for severe obesity, as they display metabolic and cardiovascular abnormalities akin to those seen in human metabolic syndrome. However, utilizing a genetic model may influence the translatability of our findings, since human polygenic obesity is not characterized by leptin deficiency, and leptin itself might overlap with the modulation of metabolic pathways affected by SGLT2i treatment. Additionally, even a relationship between leptin and FGF-21 has been hypothesized, with leptin modulating the secretion of plasma FGF-21 [36, 37], and this may further explain why our results may slightly differ from other studies [38]. However, to the best of our knowledge, this is the first report about the differential effects of SGLT2i according to the baseline energy conditions, opening new considerations to unravel in future studies and to extend our observations to different models of obesity, identifying possible implications in clinical practice.

## Data Availability

The datasets of the study are available from the corresponding author upon reasonable request.
